# Global Rate of Willingness to Volunteer Among Medical and Health Students During Pandemic: Systemic Review and Meta-Analysis

**DOI:** 10.2196/56415

**Published:** 2024-04-15

**Authors:** Mahsusi Mahsusi, Syihaabul Hudaa, Nuryani Nuryani, Mustofa Fahmi, Ghina Tsurayya, Muhammad Iqhrammullah

**Affiliations:** 1 Department of Islamic Education Management Faculty of Tarbiyah and Teacher Training Universitas Islam Negeri Syarif Hidayatullah Jakarta Tangerang Selatan Indonesia; 2 Department of Management Institut Teknologi dan Bisnis Ahmad Dahlan Jakarta Banten Indonesia; 3 Department of Indonesian Language and Literature Education Faculty of Tarbiyah and Teacher Training Universitas Islam Negeri Syarif Hidayatullah Jakarta Tangerang Selatan Indonesia; 4 Ministry of Religious Affairs of the Republic of Indonesia Jakarta Indonesia; 5 Medical Research Unit School of Medicine Universitas Syiah Kuala Banda Aceh Indonesia; 6 Postgraduate Program of Public Health Universitas Muhammadiyah Aceh Banda Aceh Indonesia

**Keywords:** COVID-19, education, health crisis, human resource management, volunteer

## Abstract

**Background:**

During health crises such as the COVID-19 pandemic, shortages of health care workers often occur. Recruiting students as volunteers could be an option, but it is uncertain whether the idea is well-accepted.

**Objective:**

This study aims to estimate the global rate of willingness to volunteer among medical and health students in response to the COVID-19 pandemic.

**Methods:**

A systematic search was conducted on PubMed, Embase, Scopus, and Google Scholar for studies reporting the number of health students willing to volunteer during COVID-19 from 2019 to November 17, 2023. The meta-analysis was performed using a restricted maximum-likelihood model with logit transformation.

**Results:**

A total of 21 studies involving 26,056 health students were included in the meta-analysis. The pooled estimate of the willingness-to-volunteer rate among health students across multiple countries was 66.13%, with an I2 of 98.99% and *P* value of heterogeneity (*P*-Het)<.001. Removing a study with the highest influence led to the rate being 64.34%. Our stratified analyses indicated that those with older age, being first-year students, and being female were more willing to volunteer (*P*<.001). From highest to lowest, the rates were 77.38%, 77.03%, 65.48%, 64.11%, 62.71%, and 55.23% in Africa, Western Europe, East and Southeast Asia, Middle East, and Eastern Europe, respectively. Because of the high heterogeneity, the evidence from this study has moderate strength.

**Conclusions:**

The majority of students are willing to volunteer during COVID-19, suggesting that volunteer recruitment is well-accepted.

## Introduction

The initial outbreak of COVID-19, an emerging and highly infectious respiratory illness, which originated in Wuhan City, Hubei Province, China, occurred in early December 2019 [1]. Subsequently, the situation escalated swiftly, leading to its declaration as a Public Health Emergency of International Concern by the World Health Organization (WHO) [1]. Some of its clinical presentations are fever, cough, dryness, fatigue, dyspnea, and myalgia [2]. The disease also induces prolonged anxiety, chest pain, persistent depression, dizziness, and other lingering symptoms after recovery [3]. In 2020, the WHO reported the rapid spread of the disease to various parts of the world, marking it as a global pandemic, with the number of confirmed cases and deaths escalating worldwide [4]. The pandemic not only affects health but also disrupts various aspects of life, including emotional stability, environmental quality, and the economy [5-7].

The high incidence of COVID-19 has led to an increased demand for health care services and workers [8]. Unlike many other sectors, jobs in health care were not temporarily halted during the COVID-19 pandemic, as health care professionals are essential in combating and preventing viral transmission [9]. However, infections among medical personnel have resulted in an acute shortage of workforce in this sector. Coupled with the increased workload of health care workers, this has led to inadequate patient management [8,10]. A previous study found that nearly half of health care workers exposed to COVID-19 experienced burnout and compassion fatigue, stemming from factors such as excessive workload, emotional exhaustion, personal infection risk, and fear of transmitting the virus to their families [5,11]. Consequently, hospitals faced the challenge of addressing staffing deficits [12].

During health emergencies, it is crucial to bolster the human resource capacity within the health care system. Among the various approaches available, recruiting volunteers is an option worthy of consideration [13]. Volunteering entails participating in activities where individuals dedicate their time to providing services to vulnerable populations without coercion [14,15]. Medical and health students can actively participate in volunteering activities to help manage the COVID-19 crisis. In certain countries and health care institutions, it is suggested that medical and health students voluntarily contribute to crisis management based on their competencies [13,16,17]. Collaborating with volunteers to provide community services could help bridge gaps in human resource capacity and decrease instances of burnout among health care workers during the COVID-19 crisis [18].

Volunteering among health care students has emerged as a valuable resource during outbreaks. A previous study has evaluated the willingness of medical students to volunteer during pandemics and disasters [19]. Furthermore, a previously published systematic review on the willingness of health students to volunteer for COVID-19 reported willingness-to-volunteer rates ranging from 19.5% to 91.5% [20]. Unfortunately, a meta-analysis was not conducted in that systematic review [20]. Data on the global rate of willingness to volunteer are necessary as a basis for evaluating the feasibility of recommending volunteering for health students. Moreover, it is crucial to observe feasibility across different populations, economies, and regions. Therefore, our aim is to conduct a new systematic review with a meta-analysis on the willingness-to-volunteer rate among medical and health students in response to the COVID-19 pandemic.

## Methods

### Study Design

The PRISMA (Preferred Reporting Items for Systematic Reviews and Meta-Analyses) statement was used as the guidance for this study (see Tables S1 and S2 in Multimedia Appendix 1) [21]. The research questions were formulated as follows: (1) What is the percentage of COVID-19 volunteer willingness among health care students? (2) What are the demographic factors associated with the willingness of health care students to volunteer? This review was not registered because it did not evaluate direct effects on human health.

### Search Strategy

A systematic review search was carried out on PubMed, Scopus, and Embase up to December 10, 2023. Google Scholar was also included as a gray literature source in the search. The keywords used were “health students,” “willingness,” “volunteer OR volunteering OR volunteerism OR voluntary,” AND “COVID-19 OR covid-19 OR SARS-CoV-2 OR COVID-19 pandemic.” The complete search technique is outlined in Table S3 in Multimedia Appendix 2 and searches for the other databases were developed using the Embase search strategy.

### Eligibility Criteria, Articles Selection, and Data Extraction

The inclusion criteria were cross-sectional studies aimed at evaluating the rate of willingness to volunteer among medical and health students (encompassing disciplines such as medical, nursing, pharmacy, dentistry, midwifery, public health, and other relevant fields) in response to the COVID-19 situation from December 2019 to December 2023. Willingness to volunteer was defined as a “yes” response to the question “Are you willing to volunteer?” or “Do you want to volunteer?” We only included studies involving undergraduate or diploma students; studies involving other levels of education were excluded. Medical and health students were defined as individuals pursuing higher education degrees (undergraduate or diploma) in medicine, nursing, dentistry, pharmacy, midwifery, public health, and related fields. Exclusion criteria were applied to studies meeting any of the following conditions:(1) qualitative analysis, (2) focused on postgraduate or professional students, (3) non–English language articles, (4) review articles, (5) case reports, (6) randomized controlled trials, (7) clinical trial proposals, and (8) case-control studies.

GT and MI independently screened all duplicate topics, titles, abstracts, and full texts using Zotero version 6.0.30 (Corporation for Digital Scholarship). Duplicate entries were removed, and title and abstract screening were conducted on the remaining records. Subsequently, the selected records were searched for full-text access, and further comprehensive screening was conducted on the obtained full texts by applying the eligibility criteria. Data extraction was conducted using the tabulation method, covering details such as author and year of publication, country, student population, sample size, gender distribution, academic year of the students, health status, marital status, living arrangements, volunteer experience, and the proportion indicating willingness. GT and MI independently carried out the data extraction process. Continuous data were presented as mean (SD), with conversion from median performed when necessary using an online calculator [22]. Any discrepancies were resolved through consensus.

### Quality Appraisal

The quality of individual studies was assessed by one reviewer (MM) and independently reviewed for agreement by a second reviewer (SH). In instances of disagreement, a third review author (NN) was consulted. The standardized Quality Assessment Checklist for Survey Studies in Psychology (Q-SSP) tool, consisting of 20 checklist items, was used for the quality assessment of the included studies [23]. High-quality articles were defined as those scoring 70% or higher. The score was determined by the percentage of “yes” responses on the checklist. The utilization of this tool aligns with a previous study [24].

### Statistical Analysis

The proportion was initially transformed using the logit function (y=logit[x]) before being pooled for meta-analysis with a restricted maximum-likelihood model. The rate was then obtained by multiplying the pooled proportion, following the back transformation from the logit function (y=1/(1+exp[–x]), with 100%. A rate exceeding 50% was considered the threshold for determining the majority’s willingness to volunteer during the pandemic. The CI was set at 95% (ie, 95% CI), with a *P* value of total effect (*P*-tot) <.05 indicating statistical significance. A value of I^2^ greater than 50% or a *P* value of heterogeneity (*P*-Het) <.1 was used as the cutoff for determining data heterogeneity in the pooled analysis. Begg’s funnel plot was used to assess the presence of publication bias. The meta-analysis was conducted using jamovi 2.3.21. A moderator analysis was conducted to examine the effects of sample size, age, gender (indicated as male-to-female ratio), academic year (indicated as the ratio of students in second to first year, third to first year, and so on), volunteer experience (indicated as the ratio of students with to without volunteer experience), type of academic program (indicated as the ratio of medical to nursing students and the ratio of medical to dentistry students), country income category, and continent. Country income was categorized based on the World Bank classifications (high income, upper middle income, lower middle income, and low income). The variables used in the moderator effect analysis were also used in the stratification analysis, with the following cutoffs: 22 years old for age, 1 for the male-to-female ratio, 15% for the proportion of first-year students, 1 for the ratio of students with to without volunteer experience, and 5 for the ratio of medical to nursing students. Statistical significance in the stratification analysis was determined using Z-statistics. The statistical analysis adhered to recommendations from previous studies [24-26].

## Results

### Search Findings

**Figure 1:**
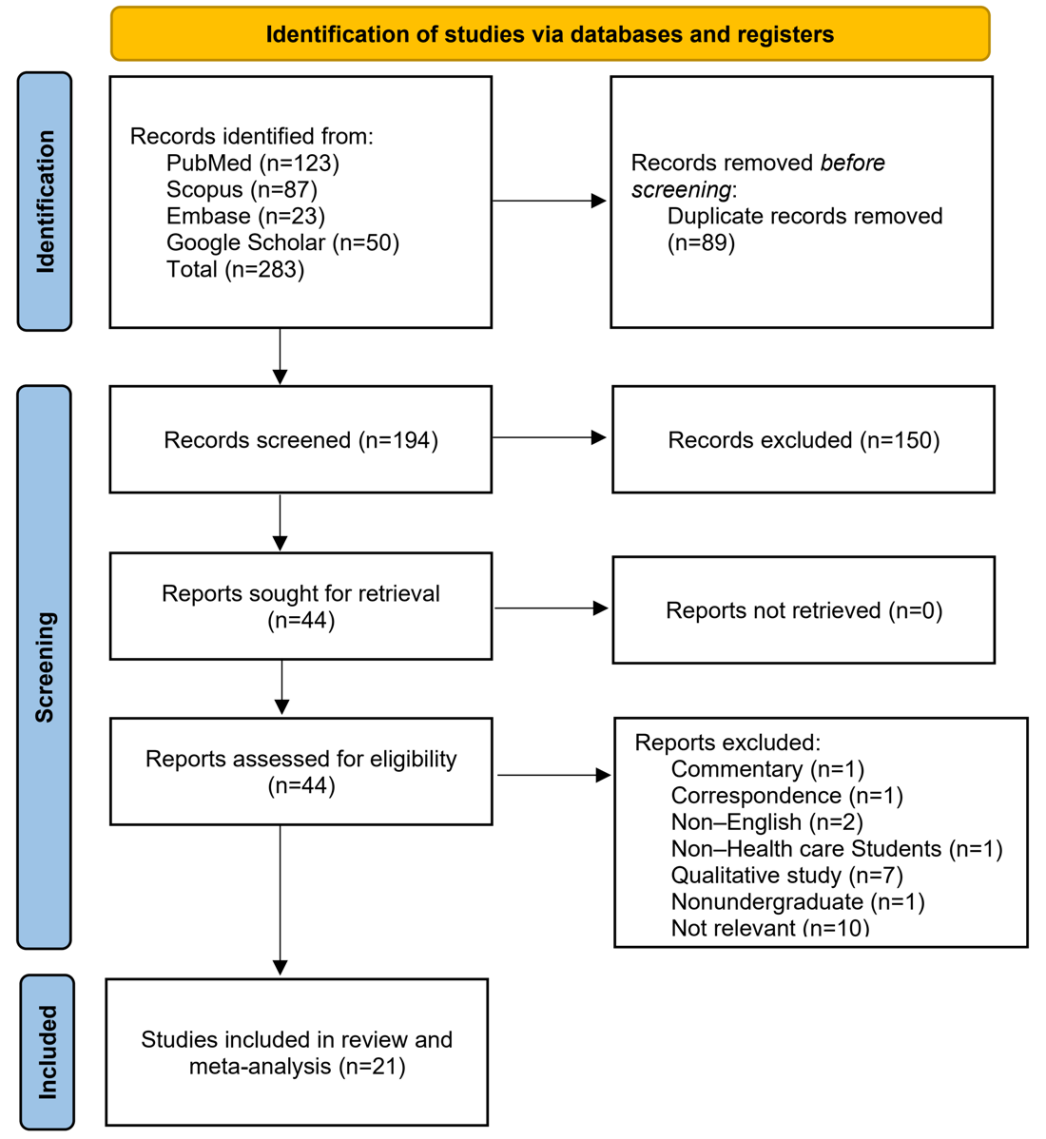
Schematic diagram for screening and selection of eligible studies following PRISMA guideline. PRISMA: Preferred Reporting Items for Systematic Reviews and Meta-Analyses.

Collectively, 283 records were identified from PubMed, Scopus, Embase, and Google Scholar in the initial stage. A total of 89 duplicates were automatically detected and subsequently removed. The remaining 194 records underwent screening for relevance based on the title and abstract. Forty records were then selected for full-text access and further thorough screening. During this stage, we identified 1 commentary [27], 1 correspondence [28], and 2 non-English articles [29,30], which were subsequently excluded. One study was excluded because the participants were not specified as medical or health students [31]. Seven studies were found to be qualitative, and therefore data extraction was not feasible; these studies were subsequently removed [32-38]. Additionally, 1 study was excluded because the participants were not pursuing undergraduate degrees [39]. Ten studies were deemed irrelevant to the objective of this review [40-49]. Finally, 21 studies were included in the systematic review and meta-analysis [50-70]. The screening and selection processes are depicted in Figure 1.

### Characteristics and Quality of the Included Studies

Characteristics of the included studies along with their quality are presented in Table 1. A total of 21 studies were included, with a combined sample size of 26,024 students [50-70]. The studies were conducted in various countries, including Nigeria (n=3), Pakistan (n=2), Saudi Arabia (n=2), Serbia (n=1), India (n=1), Bulgaria (n=1), Vietnam (n=1), Poland (n=1), Brunei Darussalam (n=1), Australia (n=1), Nepal (n=1), Indonesia (n=1), Romania (n=1), the United Kingdom (n=1), China (n=1), Syria (n=1), and Sudan (n=1). Eligible studies from South and North Americas (including the United States, Canada, and Mexico) were not identified in this systematic review. The average ages of the participants ranged from 22 to 24 years, whereas the proportion of female students varied considerably across studies. Nine studies exclusively recruited medical students [50-54,61,62,67,69], 3 focused on nursing students [55,60,65], and others included a mix of students from different departments. Thirteen studies were categorized as “high quality” based on the Q-SSP [50-62], while others had scores below 70% [63-67], and some even scored 50% or below [68-70]. Detailed assessment results based on the Q-SSP tool are presented in Table S4 in Multimedia Appendix 2 [50-70]. A total of 9/21 (43%) studies did not provide sufficient justification for the sample size. See Table S5 in Multimedia Appendix 2 for the 20 checklist items of Q-SSP and their respective code.

**Table 1:** Characteristics and quality of the included studies.

Author [reference]	Country	Geographic location	Female, n (%)	Age (years), mean (SD)	Department or faculty	Q-SSP^a^, %
Byrne et al [67]	United Kingdom	Western Europe	835 (72.9)	22 (0.61)	Medicine	60
Gazibara and Pesakovic [62]	Serbia	Eastern Europe	247 (75.8)	23.0 (1.2)	Medicine	75
Yordanova et al [68]	Bulgaria	Eastern Europe	Not reported	Not reported	Medicine, nursing, physician assistant, medical rehabilitation and occupational therapy, and midwife	45
Adejimi et al [63]	Nigeria	Africa	211 (62.6)	23.4 (2.6)	Medicine and dentistry	65
Joseph and Manasvi [69]	Indian	South Asia	119 (58.3)	21.6 (1.1)	Medicine	50
Nazir et al [61]	Pakistan	South Asia	77 (27.3)	21.9 (1.26)	Medicine	70
Tran et al [64]	Vietnam	East and Southeast Asia	1192 (58.7)	22.8 (3.7)	General medicine, traditional medicine, pharmacy, medical technique, preventive medicine, nursing, dentistry, public health, midwifery, and medical imaging	65
Domaradzki and Walkowiak [59]	Poland	Western Europe	116 (27.7)	Not reported	Medicine, nursing, midwife, pharmacy, electroradiology, medical analytics, dentistry, medical rescue, and others	70
Hj Abdul Aziz et al [60]	Brunei Darussalam	East and Southeast Asia	16 (22.2)	Not reported	Nursing	80
Prisca et al [65]	Nigeria	Africa	598 (82.6)	21.5 (2.5)	Nursing	65
Adejimi et al [58]	Nigeria	Africa	257 (62.5)	23.26 (2.59)	Medicine and dentistry	80
Al Gharash et al [55]	Australian	Pacific	5 (5.6)	Not reported	Nursing	75
AlOmar et al [56]	Saudi Arabia	Middle East	3506 (58.3)	22.07 (1.84)	Medicine, nursing, dentistry, applied medical sciences, and public health	90
Karki et al [57]	Nepal	South Asia	152 (58.2)	Not reported	Medicine and nursing	90
Khalid et al [53]	Pakistan	South Asia	142 (71.0)	21.5 (1.4)	Medicine	85
Lazarus et al [54]	Indonesia	East and Southeast Asia	3399 (69.8)	20 (0.27)	Medicine	80
Magdas et al [70]	Romania	Eastern Europe	805 (78.8)	Not reported	Medicine and nursing	50
Feng et al [66]	China	East and Southeast Asia	3582 (66.6)	20 (1.5)	Medicine, nursing, public health, medical technology, and health and medical administrative services	60
AlSaif et al [50]	Saudi Arabia	Middle East	39 (29.1)	Not reported	Medicine	75
Alsuliman et al [51]	Syria	Middle East	589 (49.1)	Not reported	Medicine	95
Elsheikh et al [52]	Sudan	Africa	424 (68.2)	23 (2)	Medicine	80

^a^Q-SSP: Quality Assessment Checklist for Survey Studies in Psychology.

### Willingness-to-Volunteer Rate

**Figure 2:**
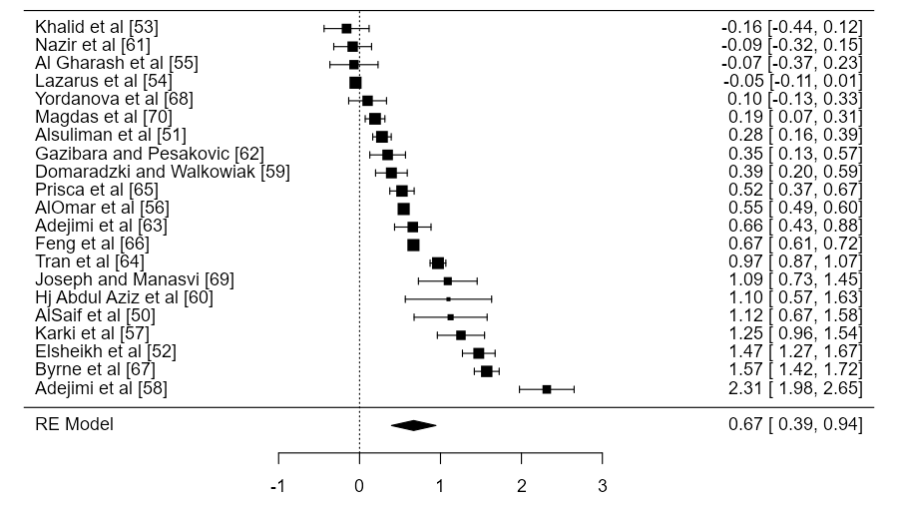
Forest plot for the pooled proportion of willingness to volunteer among health students. RE: random effect.

The forest plot of the pooled analysis on the rate of willingness to volunteer is presented in Figure 2. After being transformed back from the logit function, the pooled proportion of willingness to volunteer was 66.13% (95% CI 56%-72%). The heterogeneity for this pooled estimate was high, with I^2^=98.99% and *P*-Het<.001.

### Sensitivity Analysis

To observe if a single study affects the entire pooled estimate, a sensitivity test based on a one-leave-out analysis was conducted. The pooled estimates for each study removed are presented in Table 2. The lowest logit proportion was obtained when Adejimi et al [58] was removed (0.59, 95% CI 0.35-0.82), with the I^2^ value becoming relatively lower (72.06%), although the *P*-Het remained <.001. The overall rate of willingness to volunteer after the removal of Adejimi et al [58] was 64.34% (95% CI 59%-69%). It is noteworthy that the rate of willingness to volunteer reported by Adejimi et al [58] was the highest among all included studies, at 90.97%.

**Table 2:** Results from the one-leave-out analysis for the pooled willingness to volunteer among health students.

Study removed	Logit proportion	95% CI	*P* value of total effect	I^2^, %	*P* value of heterogeneity
Hj Abdul Aziz et al [60]	0.65	0.37-0.94	<.001	99.06	<.001
Prisca et al [65]	0.68	0.39-0.97	<.001	99.05	<.001
Adejimi et al [58]	0.59	0.35-0.82	<.001	72.06	<.001
Adejimi et al [63]	0.67	0.38-0.96	<.001	99.07	<.001
Al Gharash et al [55]	0.71	0.43-0.98	<.001	99.01	<.001
AlOmar et al [56]	0.68	0.39-0.97	<.001	98.78	<.001
AlSaif et al [50]	0.65	0.36-0.93	<.001	99.06	<.001
Alsuliman et al [51]	0.69	0.40-0.98	<.001	99.00	<.001
Byrne et al [67]	0.62	0.35-0.89	<.001	98.93	<.001
Domaradzki and Walkowiak [59]	0.68	0.40-0.97	<.001	99.06	<.001
Gazibara and Pesakovic [62]	0.69	0.40-0.97	<.001	99.06	<.001
Joseph and Manasvi [69]	0.65	0.36-0.93	<.001	99.06	<.001
Karki et al [57]	0.64	0.36-0.92	<.001	99.03	<.001
Khalid et al [53]	0.71	0.43-0.99	<.001	98.99	<.001
Lazarus et al [54]	0.71	0.43-0.99	<.001	98.73	<.001
Magdas et al [70]	0.69	0.41-0.98	<.001	99.00	<.001
Nazir et al [61]	0.71	0.43-0.99	<.001	99.00	<.001
Tran et al [64]	0.65	0.37-0.94	<.001	98.99	<.001
Yordanova et al [68]	0.70	0.42-0.98	<.001	99.03	<.001
Feng et al [66]	0.67	0.38-0.96	<.001	98.82	<.001
Elsheikh et al [52]	0.63	0.35-0.90	<.001	98.97	<.001

### Moderator Effect

The effects of moderators were analyzed, and the results are presented in Table 3. The ratio of third- to first-year students significantly affects the overall rate of willingness to volunteer with *P*=.02. Furthermore, the higher statistical significance of the moderator effect was observed on the ratios of fourth-, fifth-, or sixth- to first-year students (*P*<.001, respectively). However, other variables did not moderate the pooled estimate of the willingness-to-volunteer rate (*P* value ranged from .22 to .70).

**Table 3:** Moderator effect on the pooled estimates of willingness-to-volunteer proportion (N=21).

Moderator	Data type	Study, n (%)	Z	*P* value
Sample size	Continuous	21 (100)	–0.61	.54
Age	Continuous	14 (67)	0.79	.43
Male-to-female ratio	Continuous	20 (95)	–1.23	.22
Second-to-first-year students ratio	Continuous	7 (33)	–2.93	.003^a^
Third-to-first-year students ratio	Continuous	7 (33)	–2.28	.02^b^
Fourth-to-first-year students ratio	Continuous	7 (33)	–3.33	<.001^a^
Fifth-to-first-year students ratio	Continuous	6 (29)	–2.27	<.001^a^
Sixth-to-first-year students ratio	Continuous	4 (19)	–4.01	<.001^a^
Single-to-married ratio	Continuous	6 (29)	–0.923	.36
With-to-without volunteer experience ratio	Continuous	9 (43)	0.946	.34
Medical-to-nursing student ratio	Continuous	7 (33)	0.671	.50
Medical-to-dentistry student ratio	Continuous	5 (24)	–0.421	.67
Country income	Category	21 (100)	0.378	.70
Continent	Category	21 (100)	–1.16	.24

^a^Significant at *P*<.01.

^b^Significant at *P*<.05.

### Stratification Analysis

We further stratified the pooled estimate of the willingness-to-volunteer rate based on several variables, and the results are presented in Table 4. According to Z-statistics, groups with older mean age, a higher number of male participants, and a higher number of first-year students had a significantly higher rate of willingness to volunteer (*P*<.001). Conversely, a higher number of participants from medical school contributed to a lower rate of willingness to volunteer (*P*<.001). As compared with the pooled rates of willingness to volunteer in high-income countries, those in upper-middle-income (*P*<.001) and low-income countries (*P*=.04) tend to be significantly lower, except for lower-middle-income countries (66.37% vs 69.42%; *P*<.001). Based on regions, rates of willingness to volunteer were the highest among African and Western European countries (77.38% and 77.03%, respectively). It is worth noting that the heterogeneity of a pooled estimate of studies from Eastern European countries was negligible (I^2^=0.05%, *P*=.30), where the rate was 55.23%—the lowest among all regions. The number of samples recruited in studies according to regions and their corresponding rate of willingness to volunteer are presented in Multimedia Appendix 3.

**Table 4:** Stratification analysis based on the characteristics of participants.

Variable			Study, n (%)		Sample, n		Logit proportion		95% CI		Rate, %		*P*-Z		I^2^, %		*P* value of heterogeneity
**Mean age (years)**																	
	≤22	7 (33.3)		12,757		0.51		0.02 to 0.99		62.41		<.001^a^		98.97		<.001	
	>22	6 (21.57)		9744		1.04		0.47 to 1.62		73.89		N/A^b^		99.08		<.001	
**Male-to-female ratio**																	
	≤1	14 (66.66)		23,528		0.81		0.45 to 1.16		69.21		<.001^a^		99.31		<.001	
	>1	6 (21.57)		2246		0.42		0.01 to 0.83		60.34		N/A		94.12		<.001	
**Proportion of first-year students**																	
	≤15	3 (14.28)		1665		0.16		–0.14 to 0.45		54		<.001^a^		86.11		.006	
	>15	4 (19.04)		3510		1.23		0.94 to 1.53		77.38		N/A		88.5		<.001	
**With-to-without volunteer experience ratio**																	
	≤1	5 (23.80)		7524		0.56		–0.14 to 1.26		63.65		.156		99.22		<.001	
	>1	4 (19.04)		2833		0.63		0.17 to 1.10		65.25		N/A		95.25		<.001	
**Medical-to-nursing student ratio**																	
	≤5	3 (14.28)		5922		0.67		0.03 to 1.34		66.15		<.001^a^		97.08		<.001	
	>5	4 (19.04)		9513		0.53		0.20 to 0.85		62.95		N/A		97.6		<.001	
**Country income**																	
	High income	7 (33.33)		8974		0.68		0.24 to 1.12		66.37		Reference		98.15		<.001	
	Upper middle income	4 (19.04)		10,857		0.27		–0.05 to 0.59		56.71		<.001^a^		97.95		<.001	
	Lower middle income	8 (38.09)		4404		0.82		0.27 to 1.36		69.42		<.001^a^		97.94		<.001	
	Low income	2 (9.52)		1821		0.57		0.47 to 0.67		63.88		.041^c^		99.02		<.001	
**Regions**																	
	Africa	4 (19.04)		2096		1.23		0.54 to 1.93		77.38		Reference		97.64		<.001	
	Eastern Europe	3 (14.28)		1656		0.21		0.12 to 0.30		55.23		<.001^a^		0.05		.30	
	Western Europe	2 (9.52)		1562		1.21		1.00 to 1.24		77.03		.802		98.84		<.001	
	East and Southeast Asia	4 (19.04)		12,353		0.64		0.20 to 1.08		65.48		<.001^a^		99.08		<.001	
	South Asia	4 (19.04)		898		0.52		–0.12 to 1.16		62.71		<.001^a^		94.97		<.001	
	Middle East	3 (14.28)		7317		0.58		0.24 to 0.91		64.11		<.001^a^		94.95		<.001	

^a^Significant at *P*<.01.

^b^N/A: not applicable.

^c^Significant at *P*<.05.

### Publication Bias

**Figure 3:**
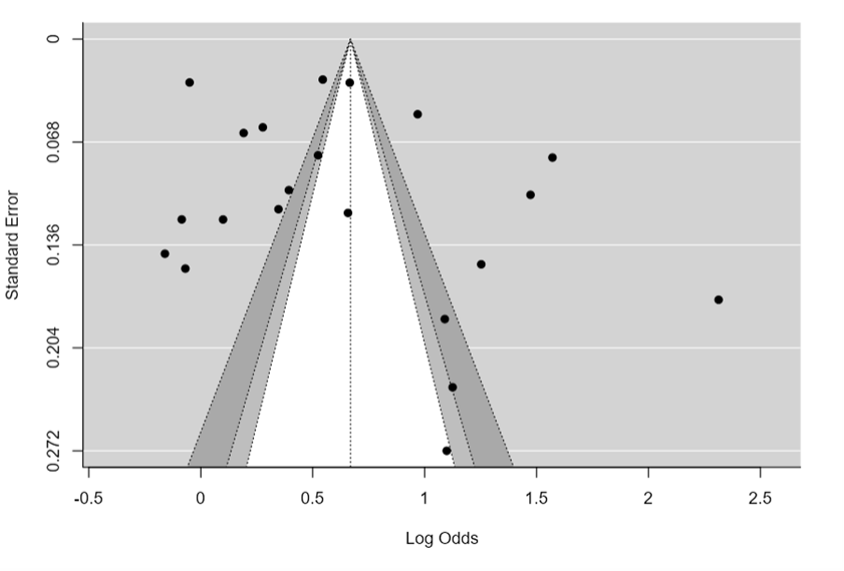
Begg’s funnel plot for studies reporting willingness to volunteer during pandemic among health students. The shape is symmetrical with *P*-Begg’s=.14.

Begg’s funnel plot for the overall pooled estimate is presented in Figure 3. The symmetrical shape of the funnel plot suggests that publication bias was not detected, with *P*-Begg=.14.

## Discussion

### Rate of Willingness to Volunteer During the Pandemic

The pooled estimate herein revealed that the overall rate of willingness to volunteer was 66.13%, with the rate being over 50% in almost every individual study. When stratified based on regions, the highest rate of willingness to volunteer was found among students from African countries (77.38%), followed by Western European countries (77.03%). Furthermore, the stratified analyses indicate that being older or female was associated with a higher rate of willingness to volunteer (*P*<.001, respectively). In addition, students in the first academic years were more willing to volunteer compared with those in more senior years (*P*<.001). According to individual studies, students were willing to volunteer due to various motivations, particularly internal factors [50-70]. Studies reported that students are driven by altruistic and duty-driven reasons, where individuals, particularly those aspiring to become future health professionals, feel a sense of responsibility in assisting during the pandemic [35]. They also perceive volunteering activity as an opportunity to learn, gain clinical skills, and enhance personal growth [34]. Research suggests that medical students exhibiting high prosocial motivation are more likely to engage in volunteer activities and persist in such endeavors, even in the absence of prior experience [49]. Moreover, external motivations contribute to such willingness in various forms, notably compensation-related factors. These external motivations include the recognition of academic credit, achievements, receipt of scholarships, and provision of material compensation [49]. Importantly, the willingness of students to volunteer is heightened when such engagement aligns with governmental needs and is endorsed by universities. Students are more likely to engage in volunteering if it is needed by the government and recommended by universities [71].

Despite the positive aspects of volunteering, attention should also be given to the mental health of students. A previous study by Tempski and colleagues [16] revealed a negative association between the mental health problems of medical students (including stress, anxiety, and depression) and their participation as volunteers during the COVID-19 pandemic. Furthermore, many volunteer students express fear of getting infected and spreading the virus to their relatives or friends during their volunteer tasks [72,73]. Students also reported feeling unprepared to deal with the pandemic, citing issues such as personal protective equipment shortages, lack of training and knowledge, role confusion, insufficient information, and a lack of support from social or family networks [71]. As highlighted in a qualitative study, the well-being of volunteers was neglected due to a lack of access to psychological support [36]. Moreover, these challenges are further perpetuated by the academic workload and responsibilities as undergraduate students [74].

In this study, we found that the willingness-to-volunteer rate among first-year students was higher compared with students in the second to sixth year of education. As suggested by a previous study, most first-year students have not yet commenced their involvement in extracurricular activities and are still in the process of selecting the type of activities they wish to pursue [75]. This further poses a challenge in using student volunteers to overcome the health care workforce shortage, as first-year students lack skills and experience, making them unsuitable for direct clinical assistance. For first-year students, community-based work is more suitable, including but not limited to childcare for health care workers, delivery of medicines to vulnerable populations, mental health checks on children, and other similar tasks. However, aiming to reduce the workload and burnout incidence among health care workers still necessitates recruiting students in higher academic years. Students in higher academic years are more involved in extracurricular activities and more occupied with lecture schedules, making them less willing to volunteer. Moreover, as students are exposed to more medical and health knowledge, they become more considerate of preventive measures. A previous study reported that students were more willing to volunteer if they were assured their grades would not suffer and be compensated, guaranteed coverage of treatment costs if they got infected while volunteering, offered separate accommodation during the duration of their volunteer work, and provided with psychological support [53]. Therefore, addressing these barriers is crucial to encourage students in higher academic years to volunteer during the pandemic.

Herein, we found that female students are more likely to volunteer than male students. This aligns with previous findings, showing a willingness proportion of 60.2% for females compared with 52.3% for males [20]. Consistent with a previous study, women were reported to be more willing to volunteer due to a nurturing inclination to help people in need and their empathetic nature, driven by personal and thoughtful motivations in the long term [76]. However, a study by Lazarus et al [54] stated that being male was one of the significant demographic factors influencing willingness to volunteer in Indonesia. The differing findings among these studies are indicative of the influence of sociocultural factors on students’ willingness to volunteer during the pandemic.

Our findings indicate that the highest rate of willingness to volunteer is observed in Africa and Western Europe, while the lowest is in Eastern Europe. This aligns with a study revealing significantly lower volunteer rates in Eastern Europe compared with Western Europe, except for the trade union [77]. One of the primary factors contributing to the lower willingness-to-volunteer rate in Eastern Europe is the historical context—having been under communist rule for half a century, memories of mandatory volunteering have imbued the concept of volunteer work with a distinctly negative connotation. This negative perception is further compounded by a postcommunist lack of trust in any public activity [78]. Nowadays, Eastern European countries have progressed beyond acknowledging volunteering to establish a legal framework that actively promotes volunteering [78]. This indicates that certain countries might have to put extra effort into encouraging students to volunteer.

In pandemic settings such as COVID-19, health students play essential roles in addressing the shortage of health care workers and responding to health problems [71]. The students’ activities can be placed into various categories, such as hospital works (triage, admission wards, and emergency rooms), call centers, administrative epidemiological aspects (contact tracing, testing), online or remote consultation (regarding COVID-19 or non–COVID-19 cases, using the phone or internet), laboratory-related works, food or personal protective equipment supply, mentoring juniors, providing childcare for health workers, public education (such as countering hoaxes), and research programs [20,45,71]. With the help of volunteers, health care providers express appreciation for their valuable contributions. There are many significant advantages to volunteering, including helping provide more services and clinical care, reducing the workload for local staff, improving the quality of care, and shortening waiting times for patients. In return, it enhances how the community views and uses health care services [79].

### Recommendations and Considerations

Based on the findings of this study, we propose several recommendations to increase the willingness-to-volunteer rate among medical and health students during the pandemic. First, we suggest implementing a robust encouragement program that integrates volunteering activities into curricula and offers psychological and accommodation support. Second, schools should prioritize the provision of high-quality training, promote knowledge, ensure clear role distribution, and effectively disseminate information to enhance the overall volunteering experience. Third, it is imperative for schools to ensure the complete safety of health care students by implementing measures such as preventing shortages of personal protective equipment, facilitating grade conversion, and guaranteeing coverage of treatment costs in case of infections incurred during volunteering.

Last but not least, although deploying students as volunteers could help overcome the health care worker shortage during the pandemic, it is important to consider potential drawbacks. The risks of contracting the disease and consequently experiencing death or long COVID-19 symptoms are high, especially during the early stages of the pandemic when managing the disease is significantly challenging. This further implicates liability issues for universities, colleges, or academic health centers. Therefore, it is crucial to actively inform students who are willing to volunteer regarding the aforementioned risks. Moreover, during volunteering, students might not be able to study optimally. The additional burden on health care workers to supervise volunteers should also be considered, implying the necessity to prepare students with volunteering skills beforehand and to establish a specific body tasked with managing the volunteers.

### Limitations

Our study is the first to calculate the global rate of willingness to volunteer during the pandemic among medical and health students. We obtained data from countries across different regions, namely, Africa, Eastern Europe, Western Europe, East and Southeast Asia, South Asia, and the Middle East. However, the study has several limitations, including being unable to retrieve data from sources other than scientific publications. We did not collect data from reports published by government or nongovernmental organizations. Additionally, we did not contact experts who might have unpublished data regarding the willingness-to-volunteer rate. The rate was calculated from heterogeneous data, which indicates the moderate strength of evidence. More than 40% of the included studies did not sufficiently justify the sample size, raising caution about the representativeness of the data. Moreover, moderator effects might be influenced by confounding factors that could not be controlled in this study. For example, as the effect of gender was observed based on the male-to-female ratio, the numbers might be influenced by differences in baseline demographics across countries and the composition of medical and other health professional schools. It is, therefore, important to confirm the findings through primary research.

### Conclusions

The overall rate of willingness to volunteer among medical and health students during COVID-19 was 66.13%. This number indicates that the recommendation for medical and health students to volunteer can be pursued, as the majority of students are willing to volunteer, although efforts to increase willingness remain necessary. Higher rates of willingness to volunteer were observed among studies with more first-year students and female participants. According to the region, students from African and Western European countries were more willing to volunteer during the pandemic. Unfortunately, the interpretation of the pooled estimate is limited by high heterogeneity, which is expected due to the variability in different countries, settings, and populations. However, this study can serve as a basis for managing medical and health students in volunteering during health crises.

## Appendix

Multimedia Appendix 1 PRISMA 20-item checklist and PRISMA abstract checklist.

Multimedia Appendix 2 Search strategy and combination of keywords used in each database; detailed assessment of the included studies using the Q-SSP tool; and the 20 checklist items of Q-SSP and their respective code. Q-SSP: Quality Assessment Checklist for Survey Studies in Psychology.

Multimedia Appendix 3 (A) Number of samples and (B) rate of willingness-to-volunteer based on regions.

## Abbreviations

*P*-Het: *P* value of heterogeneity
PRISMA: Preferred Reporting Items for Systematic Reviews and Meta-Analyses
P-tot: *P* value of total effect
Q-SSP: Quality Assessment Checklist for Survey Studies in Psychology
WHO: World Health Organization

## Data Availability

All data underlying the results are available as part of the article and no additional source data are required.
