# Supplementary material for: Global Rate of Willingness to Volunteer Among Medical and Health Students During Pandemic: Systemic Review and Meta-Analysis
Source: JMIR Med Educ. 2024 Apr 15;10:e56415. doi: 10.2196/56415 (PMC11019965; doi:10.2196/56415)
Supplement: Multimedia Appendix 2 [file mededu_v10i1e56415_app2.docx]

# **Supplementary 2**

Table S3. Search strategy and keywords combination used in each database

| Database | Keywords combination |
| --- | --- |
| PubMed | (Healthcare Students OR Health students OR Medical Students OR Nursing Students OR Dentistry Students OR Pharmacy Students OR Midwifery Students OR Health Occupations Students OR Undergraduate students OR Students) AND (Volunteers OR Voluntary Workers) AND (Willingness OR Motivation OR Involvement OR Acceptance OR Acceptability) AND (COVID 19 OR COVID-19 OR coronavirus OR Coronavirus Disease 2019 OR Wuhan Coronavirus OR SARS-CoV-2 OR 2019-ncov OR cov-19) |
| Embase | ('healthcare students':ti,ab,kw OR 'health students':ti,ab,kw OR 'medical students':ti,ab,kw OR 'nursing students':ti,ab,kw OR 'densitry students':ti,ab,kw OR 'pharmacy students':ti,ab,kw OR 'midwifery students':ti,ab,kw OR 'health occupations students':ti,ab,kw OR 'undergraduate students':ti,ab,kw OR students:ti,ab,kw) AND (volunteers:ti,ab,kw OR 'voluntary workers':ti,ab,kw) AND (willingness:ti,ab,kw OR motivation:ti,ab,kw OR involvement:ti,ab,kw OR acceptance:ti,ab,kw OR acceptability:ti,ab,kw) AND ('covid 19':ti,ab,kw OR coronavirus:ti,ab,kw OR 'coronavirus disease 2019':ti,ab,kw OR 'wuhan coronavirus':ti,ab,kw OR 'sars cov 2':ti,ab,kw OR '2019 ncov':ti,ab,kw OR 'cov 19':ti,ab,kw) |
| Scopus | TITLE-ABS-KEY ((“Healthcare Students” OR “Health students” OR “Medical Students” OR “Nursing Students” OR “Densitry Students” OR “Pharmacy Students” OR “Midwifery Students” OR “Health Occupations Students” OR “Undergraduate students” OR Students) AND (Volunteers OR “Voluntary Workers”) AND (Willingness OR Motivation OR Involvement OR Acceptance OR Acceptability) AND (“COVID 19” OR COVID-19 OR coronavirus OR “Coronavirus Disease 2019” OR “Wuhan Coronavirus” OR SARS-CoV-2 OR 2019-ncov OR cov-19) |
| Google Scholar | allintitle: (University OR college OR school OR “Student" OR "students") AND (“Volunteer” OR "volunteers" OR “Voluntary”) AND (“COVID 19” OR "COVID-19" OR pandemic) |

Table S4. Detailed assessment of the included studies using Q-SSP tool

| **Code** | Bryne 2023 | Gazibara 2020 | Yordanova 2023 | Ademiji 2022 | Joseph 2022 | Nazie 2022 | Tran 2022 | Domaradzaki 2021 | Aziz 2021 | Adejumo 2021 | Ademiji 2021 | Al Gharash 2021 | Alomat 2021 | Karki 2021 | Khalid 2021 | Lazarus 2021 | Magdas 2021 | Yu 2021 | Alsaif 2020 | Alsuliman 2020 | Elsheikh 2020 |
| --- | --- | --- | --- | --- | --- | --- | --- | --- | --- | --- | --- | --- | --- | --- | --- | --- | --- | --- | --- | --- | --- |
| 1A | ✔ | ✔ | ✔ | ✔ | ✔ | ✔ | ✔ | ✔ | ✔ | ✔ | ✔ | ✔ | ✔ | ✔ | ✔ | ✔ | ✔ | ✔ | ✔ | ✔ | ✔ |
| 1B | ✔ | ✔ | ✔ | ✔ | ✔ | ✔ | ✔ | ✔ | ✔ | ✔ | ✔ | ✔ | ✔ | ✔ | ✔ | ✔ | ✔ | ✔ | ✔ | ✔ | ✔ |
| 1C | ✔ | ✔ | ✔ | ✔ | ✔ | ✔ | ✔ | ✔ | ✔ | ✔ | ✔ | ✔ | ✔ | ✔ | ✔ | ✔ | ✔ | ✔ | ✔ | ✔ | ✔ |
| 1D | ✖ | ✖ | ✖ | ✖ | ✖ | ✖ | ✖ | ✖ | ✖ | ✖ | ✖ | ✖ | ✖ | ✖ | ✖ | ✖ | ✖ | ✖ | ✖ | ✖ | ✖ |
| 2A | ✔ | ✔ | ✔ | ✔ | ✔ | ✔ | ✔ | ✔ | ✔ | ✔ | ✔ | ✔ | ✔ | ✔ | ✔ | ✔ | ✔ | ✔ | ✔ | ✔ | ✔ |
| 2B | ✔ | ✔ | ✖ | ✔ | ✖ | ✔ | ✔ | ✖ | ✔ | ✖ | ✔ | ✔ | ✔ | ✔ | ✔ | ✖ | ✖ | ✖ | ✖ | ✔ | ✔ |
| 2C | ✖ | ✔ | ✖ | ✔ | ✖ | ✔ | ✔ | ✖ | ✔ | ✖ | ✔ | ✖ | ✔ | ✔ | ✔ | ✔ | ✖ | ✖ | ✖ | ✔ | ✔ |
| 3A | ✖ | ✖ | ✖ | ✔ | ✖ | ✖ | ✖ | ✔ | ✔ | ✔ | ✔ | ✔ | ✔ | ✔ | ✔ | ✖ | ✔ | ✔ | ✖ | ✔ | ✔ |
| 3B | ✖ | ✖ | ✖ | ✖ | ✖ | ✖ | ✖ | ✖ | ✖ | ✖ | ✖ | ✔ | ✔ | ✔ | ✔ | ✖ | ✔ | ✔ | ✖ | ✔ | ✖ |
| 3C | ✔ | ✔ | ✖ | ✖ | ✔ | ✔ | ✔ | ✔ | ✔ | ✔ | ✔ | ✔ | ✔ | ✔ | ✔ | ✔ | ✔ | ✔ | ✔ | ✔ | ✔ |
| 3D | ✔ | ✔ | ✔ | ✔ | ✔ | ✔ | ✔ | ✔ | ✔ | ✔ | ✔ | ✔ | ✔ | ✔ | ✔ | ✔ | ✖ | ✔ | ✔ | ✔ | ✔ |
| 3E | ✔ | ✔ | ✖ | ✔ | ✔ | ✔ | ✖ | ✔ | ✔ | ✔ | ✖ | ✖ | ✔ | ✔ | ✔ | ✔ | ✖ | ✖ | ✔ | ✔ | ✖ |
| 3F | ✔ | ✔ | ✔ | ✖ | ✖ | ✖ | ✖ | ✖ | ✖ | ✖ | ✔ | ✔ | ✔ | ✔ | ✔ | ✔ | ✖ | ✖ | ✔ | ✔ | ✔ |
| 3G | ✔ | ✔ | ✔ | ✔ | ✔ | ✔ | ✔ | ✔ | ✔ | ✔ | ✔ | ✔ | ✔ | ✔ | ✔ | ✔ | ✔ | ✖ | ✔ | ✔ | ✔ |
| 3H | ✔ | ✔ | ✖ | ✔ | ✔ | ✔ | ✔ | ✔ | ✔ | ✖ | ✔ | ✔ | ✔ | ✔ | ✔ | ✔ | ✔ | ✖ | ✔ | ✔ | ✖ |
| 3I | ✖ | ✔ | ✔ | ✔ | ✖ | ✔ | ✔ | ✔ | ✔ | ✔ | ✔ | ✔ | ✔ | ✔ | ✔ | ✔ | ✖ | ✔ | ✔ | ✔ | ✔ |
| 3J | ✔ | ✔ | ✔ | ✔ | ✔ | ✔ | ✔ | ✔ | ✔ | ✔ | ✔ | ✔ | ✔ | ✔ | ✔ | ✔ | ✔ | ✔ | ✔ | ✔ | ✔ |
| 4A | ✖ | ✔ | ✖ | ✔ | ✔ | ✔ | ✔ | ✔ | ✔ | ✔ | ✔ | ✔ | ✖ | ✔ | ✔ | ✔ | ✖ | ✔ | ✔ | ✔ | ✔ |
| 4B | ✖ | ✖ | ✖ | ✖ | ✖ | ✖ | ✖ | ✖ | ✖ | ✖ | ✖ | ✖ | ✔ | ✔ | ✖ | ✔ | ✖ | ✖ | ✔ | ✔ | ✔ |
| 4C | ✖ | ✖ | ✖ | ✔ | ✖ | ✖ | ✖ | ✔ | ✔ | ✔ | ✔ | ✖ | ✔ | ✔ | ✖ | ✔ | ✖ | ✔ | ✔ | ✔ | ✔ |
| Yes (%) | 60% | 75% | 45% | 65% | 50% | 70% | 65% | 70% | 80% | 65% | 80% | 75% | 90% | 90% | 85% | 80% | 50% | 60% | 75% | 95% | 80% |

Table S5. Twenty-checklist items of Q-SSP and their respective code

| **#** | **Code** | **Domain** | **Item** |
| --- | --- | --- | --- |
| 1 | 1A | Introduction | Was the problem or phenomenon under investigation defined, described, and justified? |
| 2 | 1B | Introduction | Was the population under investigation defined, described, and justified? |
| 3 | 1C | Introduction | Were specific research questions or hypotheses stated? |
| 4 | 1D | Introduction | Were operational definitions of all study variables provided? |
| 5 | 2A | Participants | Were participant inclusion criteria stated? |
| 6 | 2B | Participants | Was the participant recruitment strategy described? |
| 7 | 2C | Participants | Was there a justification/rationale for the sample size provided? |
| 8 | 3A | Data | Was the attrition rate provided? (applies to cross-sectional and prospective studies.) |
| 9 | 3B | Data | Was a method of treating attrition provided? (applies to cross-sectional and prospective studies.) |
| 10 | 3C | Data | Were the data analysis techniques justified (i.e., was the link between hypotheses/aims/research questions and data analyses explained)? |
| 11 | 3D | Data | Were the measures provided in the report (or in a supplement) in full? |
| 12 | 3E | Data | Was evidence provided for the validity of all the measures (or instruments) used? |
| 13 | 3F | Data | Was information provided about the person(s) who collected the data (e.g., training, expertise, other demographic characteristics)? |
| 14 | 3G | Data | Was information provided about the context (e.g., place) of data collection? |
| 15 | 3H | Data | Was information provided about the duration (or start and end dates) of data collection? |
| 16 | 3I | Data | Was the study sample described in terms of key demographic characteristics? |
| 17 | 3J | Data | Was discussion of findings confined to the population from which the sample was drawn? |
| 18 | 4A | Ethics | Were participants asked to provide informed consent or assent? |
| 19 | 4B | Ethics | Were participants debriefed at the end of the data collection? |
| 20 | 4C | Ethics | Were funding sources or conflicts of interest disclosed? |
