# Supplementary figures and images for: Global Rate of Willingness to Volunteer Among Medical and Health Students During Pandemic: Systemic Review and Meta-Analysis
Source: JMIR Med Educ. 2024 Apr 15;10:e56415. doi: 10.2196/56415 (PMC11019965; doi:10.2196/56415)

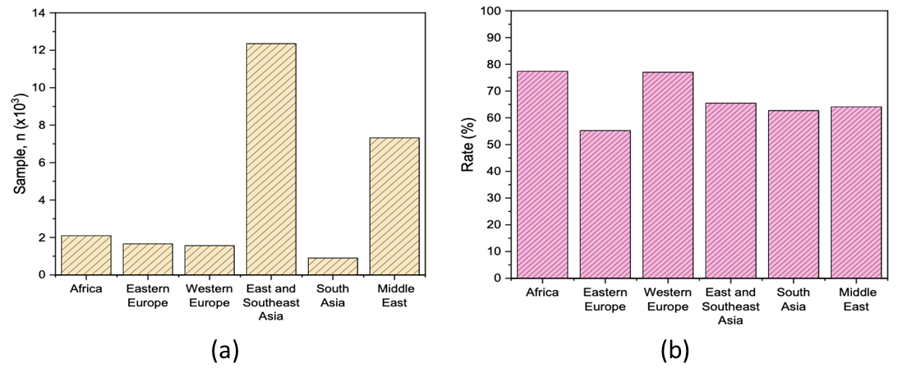

Supplement: Multimedia Appendix 3 [file mededu_v10i1e56415_app3.png]
